# Supplementary material for: Impact of B18R-Encoding Messenger Ribonucleic Acid Co-Delivery on Neutralizing Antibody Production in Self-Amplifying Messenger Ribonucleic Acid Vaccines
Source: Vaccines (Basel). 2025 May 18;13(5):537. doi: 10.3390/vaccines13050537 (PMC12115987; doi:10.3390/vaccines13050537)
Supplement: Supplementary file 1 [file vaccines-13-00537-s001.zip › vaccines-3581371-supplementary.pdf]

vaccines

MDPI

Article

Impact of B18R-Encoding Messenger Ribonucleic Acid Co-Delivery on Neutralizing Antibody Production in Self-Amplifying Messenger Ribonucleic Acid Vaccines

Yutao Wang [1,2,†], Lei Li [3,†], Min Liang [4,5], Gan Liu [5,6,7,*] and Yinying Lu [1,2,8,*]

[1] 302 Clinical Medical School, Peking University, Beijing 100039, China; 2211210746@stu.pku.edu.cn
[2] Senior Department of Hepatology, The Fifth Medical Center of PLA General Hospital, Beijing 100039, China
[3] Center for Synthetic and Systems Biology, Department of Automation, Tsinghua University, Beijing, 100084, China; lilei2022@tsinghua.org.cn
[4] Beijing Syngenbio Co., Ltd., Beijing 100176, China; m.liang@syngenbio.com
[5] Syngen-Bioimmune (Qing Dao) Co., Ltd., Qingdao 266000, China
[6] Beijing Syngentech Co., Ltd., Beijing 100176, China
[7] Department of Otolaryngology Head and Neck Surgery, Beijing Tongren Hospital, Capital Medical University, Beijing 100730, China
[8] Comprehensive Liver Cancer Centre, The Fifth Medical Center of PLA General Hospital, Beijing 100039, China
* Correspondence: g.liu@syngen.tech (G.L.); luyinying1973@163.com (Y.L.)
† These authors contributed equally to this work.

Academic Editors: Apostolos P. Georgopoulos and Ulrich Strych

Received: 27 March 2025
Revised: 8 May 2025
Accepted: 16 May 2025
Published: 18 May 2025

Citation: Wang, Y.; Li, L.; Liang, M.; Liu, G.; Lu, Y. Impact of B18R-Encoding Messenger Ribonucleic Acid Co-Delivery on Neutralizing Antibody Production in Self-Amplifying Messenger Ribonucleic Acid Vaccines. *Vaccines* **2025**, *13*, 537. https://doi.org/10.3390/vaccines13050537

Copyright: © 2025 by the authors. Licensee MDPI, Basel, Switzerland. This article is an open access article distributed under the terms and conditions of the Creative Commons Attribution (CC BY) license (https://creativecommons.org/licenses/by/4.0/).

Supplementary Figures:

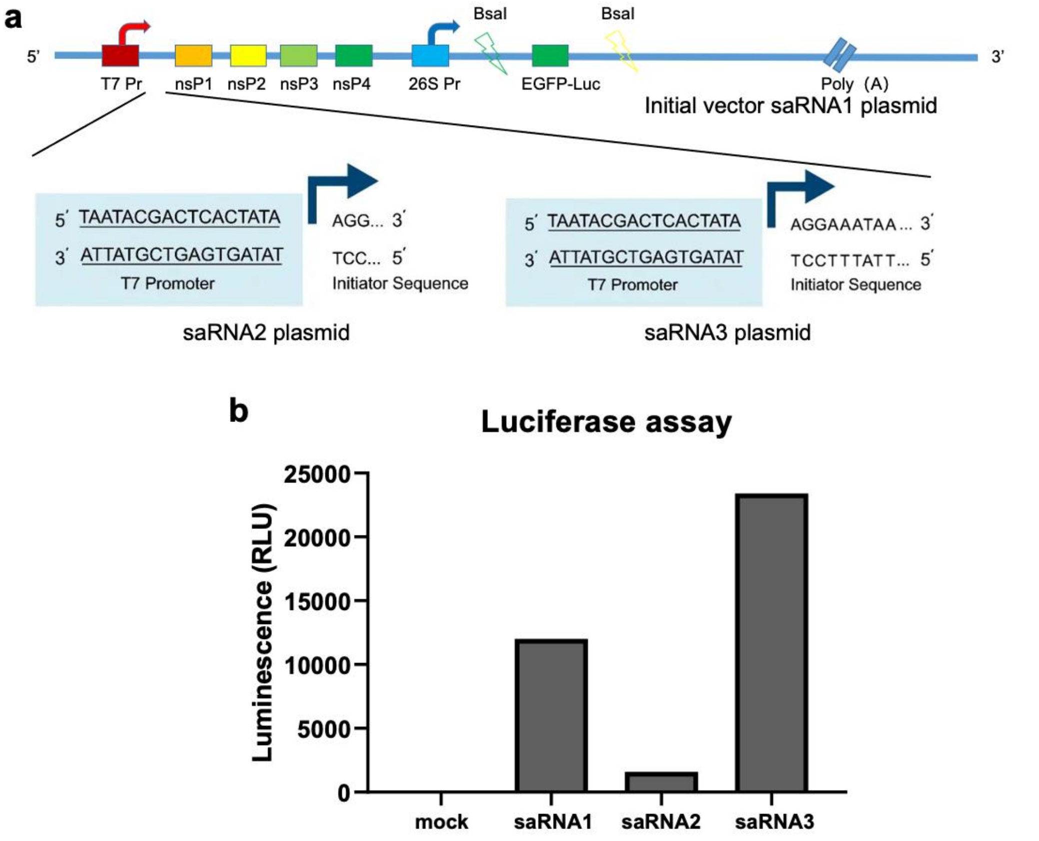

Figure S1. VEEV vector plasmid modification. a Two modification versions. b Intracellular expression of different versions of saRNA.

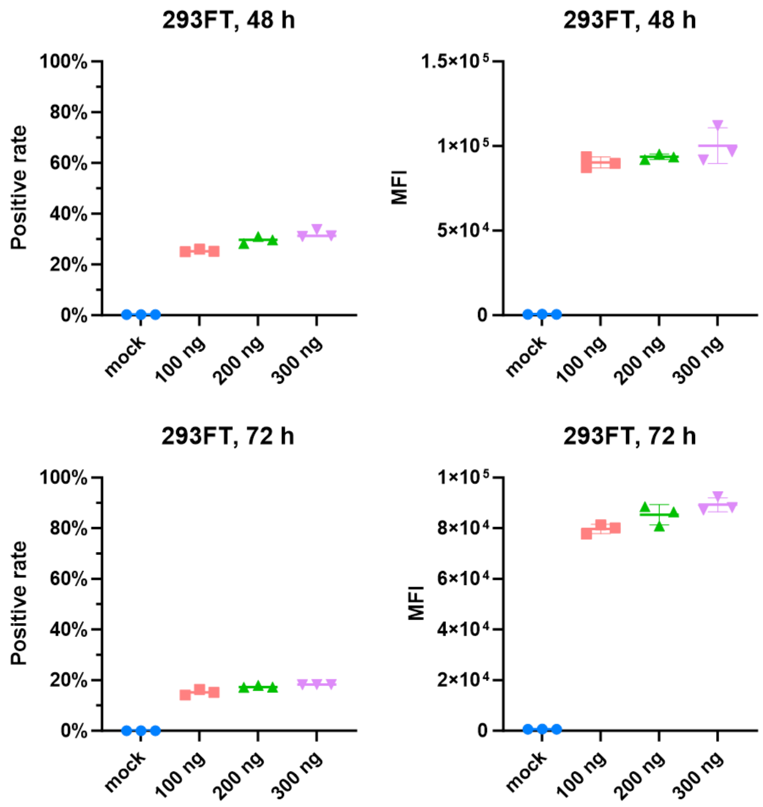

Figure S2. Transfection efficiency and MFI of varying doses of LNP-saRNA in 293FT cells.

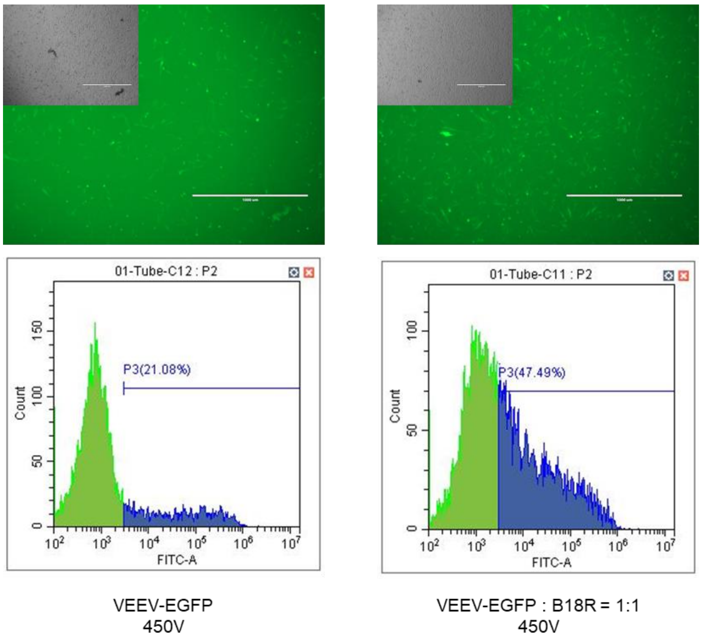

Figure S3. Microscopic images and electroporation positive rate of saRNA-EGFP after electroporation (Left). Microscopic images and electroporation positive rate following co-electroporation of saRNA-EGFP and B18R-mRNA at a 1:1 ratio (Right). Scale bar: 1000 μm.

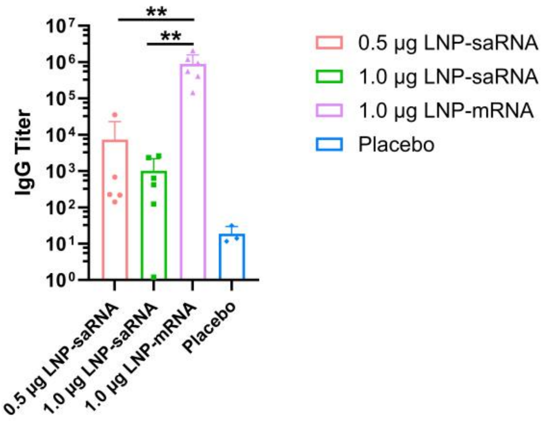

Figure S4. IgG antibody titer at day 21 post prime immunization.

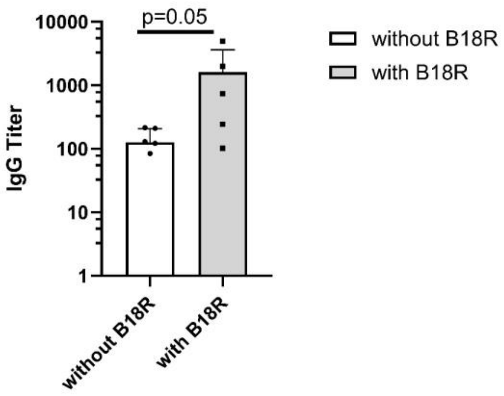

Figure S5. IgG antibody titer in the 1 µg saRNA group and the 1 µg saRNA/2 µg B18R-mRNA group.

Disclaimer/Publisher's Note: The statements, opinions and data contained in all publications are solely those of the individual author(s) and contributor(s) and not of MDPI and/or the editor(s). MDPI and/or the editor(s) disclaim responsibility for any injury to people or property resulting from any ideas, methods, instructions or products referred to in the content.
